# RNA Binding Protein OsTZF7 Traffics Between the Nucleus and Processing Bodies/Stress Granules and Positively Regulates Drought Stress in Rice

**DOI:** 10.3389/fpls.2022.802337

**Published:** 2022-02-21

**Authors:** Chiming Guo, Lingli Chen, Yuchao Cui, Ming Tang, Ying Guo, Yin Yi, Yan Li, Liqing Liu, Liang Chen

**Affiliations:** ^1^Fujian Key Laboratory of Subtropical Plant Physiology and Biochemistry, Fujian Institute of Subtropical Botany, Xiamen, China; ^2^Xiamen Key Laboratory for Plant Genetics, School of Life Sciences, Xiamen University, Xiamen, China; ^3^Key Laboratory of State Forestry Administration on Biodiversity Conservation in Karst Area of Southwestern, School of Life Sciences, Guizhou Normal University, Guiyang, China; ^4^Key Laboratory of Plant Physiology and Developmental Regulation, School of Life Sciences, Guizhou Normal University, Guiyang, China

**Keywords:** *OsTZF7*, drought, stress granules (SGs), processing bodies (PBs), nucleo-cytoplasmic trafficking, RNA-binding

## Abstract

Tandem CCCH zinc finger (TZF) proteins are the essential components of processing bodies (PBs) and stress granules (SGs), which play critical roles in growth development and stress response in both animals and plants through posttranscriptional regulation of target mRNA. In this study, we characterized the biological and molecular functions of a novel tandem zinc finger protein, OsTZF7. The expression of *OsTZF7* was upregulated by abiotic stresses, including polyethylene glycol (PEG) 4000, NaCl, and abscisic acid (ABA) in rice. Accordingly, the overexpression of *OsTZF7* increased drought tolerance and enhanced sensitivity to exogenous ABA in rice, whereas the knockdown of *OsTZF7* resulted in the opposite phenotype. RNA-seq analysis revealed that genes related to “response to stress,” “abscisic acid signaling,” “methylated histone binding,” and “cytoplasmic mRNA processing body” are regulated by *OsTZF7*. We demonstrated that OsTZF7 can traffic between the nucleus and PBs/SGs, and the leucine-rich nuclear export signal (NES) mediates the nuclear export of OsTZF7. Additionally, we revealed that OsTZF7 can bind adenine- and uridine-rich (AU-rich) element (ARE) or ARE-like motifs within the 3′ untranslated region of downregulated mRNAs, and interact with PWWP family proteins *in vitro*. Together, these results indicate that OsTZF7 positively regulates drought response in rice *via* ABA signaling and may be involved in mRNA turnover.

## Introduction

The CCCH zinc finger proteins contain one or more CCCH-type zinc finger motifs (three cysteines followed by one histidine). Notably, 68 and 67 CCCH zinc finger genes have been identified in *Arabidopsis* and rice, respectively (Wang et al., 2008). Among them, tandem CCCH zinc finger (TZF) proteins (two identical CCCH zinc fingers separated by 18 amino acids) constitute a large subfamily and have been found widely in eukaryotes (Bogamuwa and Jang, 2013).

The TZF proteins usually function as RNA binding proteins and directly bind to adenine- and uridine-rich (AU-rich) elements (AREs) within the 3′ untranslated region (3′UTR) of target mRNAs. TZFs can also recruit the CCR4-NOT complex to target mRNAs and induce mRNA decay (Otsuka et al., 2019). Another feature of TZF proteins is that they shuttle between different cellular compartments, such as from the nucleus to the cytoplasm, and between different cytoplasmic RNA granules, like polysomes, stress granules (SGs), and processing bodies (P-bodies, PBs), where they are considered to play various roles in RNA metabolism (Bogamuwa and Jang, 2014; Maldonado-Bonilla, 2014; Fu and Blackshear, 2017). In mammals, Tristetraprolin (TTP, the prototype of mammalian TZF proteins) can bind the AREs at the 3′UTR of tumor necrosis factor-α (TNF-α) and trigger TNF-α mRNA decay by recruiting deadenylation and decapping complexes. Meanwhile, the expression of *TTP* is induced by TNF-α signaling. Thus, TTP acts as a key component of a negative feedback loop that controls TNF-α production through a posttranscriptional mechanism (Carballo et al., 1998; Lai and Blackshear, 2001). The destabilization of mRNA mediated by TTP has been reported in other cytokines, such as interleukin (IL)-16, IL-8, IL-22, IL-23, interferon (IFN)-γ, granulocyte-macrophage colony-stimulating factor (GM-CSF), and some chemokines (Brooks and Blackshear, 2013).

Although TZFs have been well documented in humans and mice (Fu and Blackshear, 2017), the function of most plant TZF proteins is relatively less known. Recently, some studies suggest that TZF proteins are involved in various aspects of plant growth and development, cellular functions, and stress responses. For example, *AtTZF1, 2, 3* and *OsTZF1, 5* are involved in abiotic stresses (Huang et al., 2011, 2012; Lin et al., 2011; Lee et al., 2012; Zhang et al., 2012; Jan et al., 2013; Han et al., 2014; Selvaraj et al., 2020). *PdC3H17* depends on its CCCH domain to control drought tolerance in *Populus* (Zhuang et al., 2020). *OsTZF1* and *OsTZF2* (*OsDOS*) delay leaf senescence in rice (Kong et al., 2006; Jan et al., 2013). *AtTZF9* is involved in Pathogen-Associated Molecular Pattern (PAMP)-triggered immune response (Maldonado-Bonilla et al., 2014). In addition to stress, TZF proteins are also involved in the light signaling pathway. *AtTZF4, 5, 6* negatively regulate light-, abscisic acid (ABA)-, and gibberellic acid (GA)-mediated seed germination (Kim et al., 2008; Bogamuwa and Jang, 2013), and *OsTZF1* exhibits similar characteristics regard to light response (Zhang et al., 2012).

Mammalian TZF proteins traffic between the nucleus and cytoplasmic messenger ribonucleoprotein (mRNP) complexes (PBs and SGs). PBs and SGs are the membrane-less organelles where mRNA turnover and translational repression take place (Balagopal and Parker, 2009). The nuclear export receptor CRM1/Xpo1 mediates the nuclear export of TTP and TIS11 (mammalian TZF) by binding directly to their leucine-rich nuclear export signal (NES) (Murata et al., 2002; Phillips et al., 2002). In plants, all of the AtTZFs can localize to cytoplasmic foci (Pomeranz M. et al., 2010). AtTZF1, 9 and OsTZF1, 5 have been reported to colocalize with PBs and SGs markers and shuttle between the nucleus and cytoplasm (Pomeranz M.C. et al., 2010; Jan et al., 2013; Maldonado-Bonilla et al., 2014; Selvaraj et al., 2020). Plant TZFs are predicted to contain putative NES sequences (Bogamuwa and Jang, 2013); however, it is still unclear whether these NES sequences are functional.

In this study, we characterized the biological and molecular functions of a novel tandem zinc finger protein, OsTZF7, and demonstrated that *OsTZF7* acts as a positive regulator for drought tolerance in rice. OsTZF7 can traffic between the nucleus and PBs/SGs and rely on the C-terminal NES for its nuclear export. Additionally, RNA electrophoretic mobility shift assay (REMSA) revealed that OsTZF7 can bind to ARE or ARE-like motifs *in vitro* and interact with PWWP proteins, suggesting the possible role of OsTZF7 in mRNA metabolism.

## Materials and Methods

### Plant Materials and Stress Treatments

The japonica rice (*Oryza sativa*) cultivar (Nipponbare, Nip) was used in this study. To detect the transcript level of *OsTZF7* under various abiotic stresses and phytohormone treatment, Nip seedlings were grown in a growth chamber with a 14-h light/10-h dark cycle at 26°C. Four-leaf stage seedlings were subjected to different treatments including 20% polyethylene glycol (PEG) 4,000 (w/v), 200 mM NaCl, low temperature (4°C), and 100 μM ABA. The shoot and root tissues were sampled at 0, 1, 3, 6, 12, and 24 h after treatment.

The T3 generation of transgenic plants was used in all the experiments.

For dehydration treatment, the four-leaf stage seedlings of transgenic and Nip plants were grown hydroponically in 96-well culture boxes using Yoshida solution containing 20% (w/v) PEG4000 for 10 days. Then, the stressed plants were recovered in normal Yoshida solution for 7 days. The survival rates of transgenic lines and Nip were recorded.

To evaluate the drought tolerance of transgenic rice at the vegetative stage, the transgenic and Nip seeds were germinated on half-strength Murashige and Skoog (1/2 MS) medium with or without 50 mg/L hygromycin, respectively. Then, the positive transgenic and Nip seedlings (both with shoot height of 1 cm) were transferred to barrels. When the plants grew to the vegetative stage, the water supply was withheld for 10 days. After recovery by rewatering for a week, survival performance was photographed and recorded.

The water loss rate of detached leaves was measured using the method reported previously (Xiang et al., 2008; Mao et al., 2010). Leaves of transgenic and wild-type rice at the five-leaf stage were cut and weighed immediately and then exposed to air at room temperature and weighed every hour.

For the ABA sensitivity test, the germinated seeds were transferred in 1/2 MS medium supplemented with or without 2 μM ABA. The relative shoot length was measured after growing for 7 days.

### Plasmid Construction and Rice Transformation

To generate the *OsTZF7* overexpressing construct, the full-length coding region of *OsTZF7* (Os05g0525900) was amplified from rice cultivar Nip by reverse transcription PCR (RT-PCR). The PCR product was cloned into pCXUN under the control of the maize (*Zea mays*) ubiquitin promoter. The artificial microRNA (amiRNA) of *OsTZF7* was designed by WMD3.^1^ The amiRNA construct was generated by single-step PCR and then was cloned into pCXUN-osaMIR528 under the control of the ubiquitin promoter. For expression pattern analysis, the *OsTZF7* promoter region (2.2-kb fragment upstream of the ATG start codon) was inserted into the pCXGUS-P vector to drive the β-glucuronidase (*GUS*) reporter gene. All the constructs were introduced into Nip rice by *Agrobacterium tumefaciens*-mediated transformation (Lin and Zhang, 2005). All the primers used in this study are listed in Supplementary Table 1.

### RNA Isolation and Quantitative Real-Time PCR

Total RNA was isolated from rice using the Eastep Universal RNA Extraction Kit (Promega, United States). First-strand cDNA was synthesized from DNase I-treated total RNA using the HiScript II First-Strand cDNA Synthesis Kit (Vazyme, China). Quantitative Real-time PCR (qRT-PCR) was performed on ABI 7500 RT-PCR system (Applied Biosystems, United States) using SYBR Green Premix Pro Taq HS qRT-PCR Kit II (Accurate, China) according to the protocol of the manufacturer. Rice *UBQ5* (Os01g0328400) was used as an endogenous control. Relative expression levels were determined as described previously (Livak and Schmittgen, 2001).

### Glucuronidase Histochemical Assay

The GUS histochemical activity of pCXGUS-P/*OsTZF7* transgenic rice was detected according to the protocol described previously (Jefferson et al., 1987). Different tissues from transgenic plants were incubated in staining buffer (50 mM sodium phosphate at pH 7.0, 10 mM EDTA, 0.1% Triton X-100, 1 mg/ml X-Gluc, 100 mg/ml chloramphenicol, 1 mM potassium ferricyanide, and 1 mM potassium ferrocyanide) at 37°C overnight and then washed with 75% ethanol to remove chlorophyll. GUS images were taken with a stereomicroscope.

### Subcellular Localization and Bimolecular Fluorescence Complementation Analysis

For subcellular localization analysis, the coding sequence (CDS) of *OsTZF7* was cloned into pCXDG and fused to the green fluorescent protein (GFP) reporter gene, under the control of the CaMV35s promoter. Stable transgenic rice was generated as described above. For the colocalization studies, the CDS of *OsTZF7* was fused with GFP under the control of CaMV35s promoter in the pXDG vector. The constructs that AtDCP2 and AtPABP8, OsDCP2 and OsPABP1, 2, 3 fused with the red fluorescent protein (RFP) were generated in our previous study (Guo et al., 2016). For bimolecular fluorescence complementation (BiFC) studies, the CDS without a termination codon of *OsTZF7* or *OsPWWP1, 2, 3* was fused to the N-terminal or C-terminal fragments (YN or YC) of yellow fluorescent protein (YFP) (Shen et al., 2011), respectively. The plasmid for colocalization and BiFC were introduced into the *A. tumefaciens* strain GV3101. Tobacco transient expression assay was described previously according to the protocol (Walter et al., 2004), and the fluorescence signal was observed and photographed with a confocal microscope (LSM780; Carl Zeiss, Germany).

### RNA-Seq and Bioinformatics Analysis

Four-leaf stage seedlings were subjected to PEG4000 treatment for 12 h. Three independent biological replicates were used. Total RNA was isolated as described above. The cDNA libraries were sequenced on the Illumina sequencing platform by Gene *Denovo* Biotechnology Co., Ltd. (Guangzhou, China). The clean reads were mapped to the Rice Annotation Project Database (RAP-DB). Both log2(foldchange) ≥ 1 and false discovery rate (FDR) < 0.05 were set as the threshold of significantly differential expression. Gene Ontology (GO), pathway enrichment, dynamic gene set enrichment analysis (GSEA), and heatmap analysis were performed using the OmicShare tools, which is a free online platform for data analysis.^2^

The FIMO and MEME analysis was performed by the MEME suite (Bailey et al., 2009).

### RNA Electrophoretic Mobility Shift Assay

Recombinant protein 6 × His-SUMO-fused OsTZF7 was expressed in *Escherichia coli* strain BL21 CodonPlus (DE3) and induced with 0.5 mM isopropyl-*b*-D-thiogalactoside at 20°C overnight. Bacterial cells were collected and disrupted by sonication. The 6 × His-SUMO-OsTZF7 proteins were purified using Ni-NTA resin (Thermo Fisher Scientific, United States), and then, the 6 × His-SUMO tag was removed by on-column cleavage with Ulp1 (SUMO Protease).

pET-28a(+) plasmid containing 3′UTR of the target genes and an ARE sequence were linearized with *Bam*HI and then used for *in vitro* RNA synthesis by the RiboMAX Large Scale RNA Production System, T7 (Promega, United States). RNA probe (∼60 ng) was incubated with different concentrations of purified OsTZF7 protein in 20 μl binding buffer (10 mM Tris–HCl, pH 8.0, 40 mM KCl, 2 mM DDT, 3 mM MgCl_2_, 5 μM ZnCl_2_, 20% glycerol) at room temperature for 30 min. The RNA electrophoretic mobility shift assay (REMSA) reaction mixture was separated by 6% non-denaturing polyacrylamide gel at 4°C. The gel was then stained with SYBR Green EMSA staining solution (Thermo Fisher Scientific, United States) as described.

### Yeast Two-Hybrid Assay

The full-length OsTZF7 and OsTZF7ΔC (C-terminal NES removed) were cloned to the plasmid pGBKT7 and transformed into yeast strain Y2HGold, respectively. The transformants of the OsTZF7 and OsTZF7ΔC did not show autoactivation and toxicity. OsTZF7 or OsTZF7ΔC was used as bait to screen a rice-seedling cDNA library constructed in the pGADT7-Rec vector, respectively. Yeast two-hybrid (Y2H) assay was performed with Matchmaker Gold Yeast Two-Hybrid System according to the protocol of the manufacturer (Clontech, United States). Positive colonies were selected on SD/-Trp-Leu-His-Ade medium. After confirmation using the X-α-Gal test and retransformation, the inserts were sequenced.

## Results

### Spatial and Stress-Induced Expression Profiles of *OsTZF7*

**FIGURE 1:**
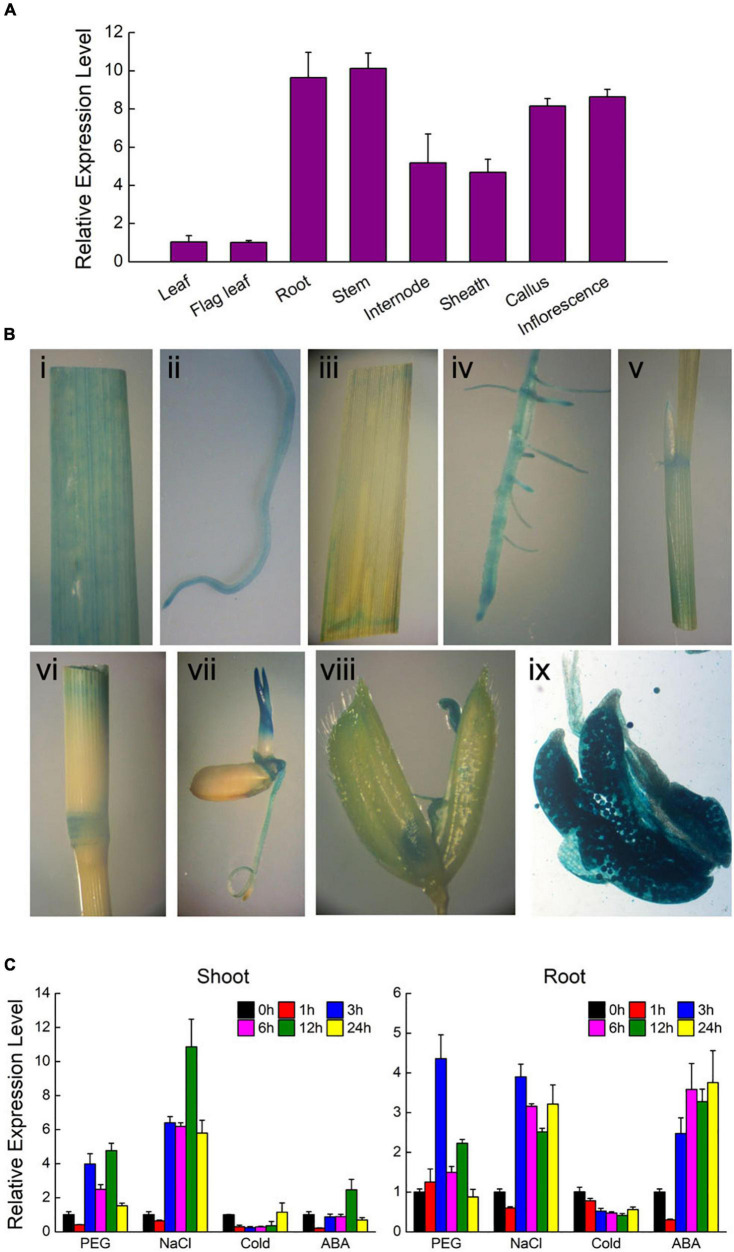
Expression profile of *OsTZF7*. **(A)** Tissue-specific expression analysis of *OsTZF7*. The relative expression level of *OsTZF7* was measured by quantitative real-time PCR (qRT-PCR). Error bars indicate SD from three replicates. **(B)** β-glucuronidase (GUS) staining in different tissues of P*_*OsTZF*7_*:*GUS* transgenic rice. *GUS* expression was detected in (i) young leaf, (ii) young root, (iii) mature leaf, (iv) mature root, (v) sheath, (vi) stem and internode, (vii) coleoptile, (viii) lemma and palea, and (ix) anther. **(C)** The relative expression level of *OsTZF7* in shoot and root under different treatments. Four-leaf stage seedlings were subjected to PEG4000 (20%), salt (200 mM NaCl), cold (4°C), and ABA (100 μM).

We performed qRT-PCR analysis to determine the expression profile of *OsTZF7* in various tissues. As shown in Figure 1A, *OsTZF7* was highly expressed in root, stem, callus, and inflorescence. To further investigate the expression profile of *OsTZF7*, the *OsTZF7* promoter fragment was fused to the *GUS* reporter gene and transformed into Nip rice. Consistent with the qRT-PCR result, strong GUS activity was detected in the young leaf, young root, mature root, stem, sheath, coleoptile, pistil, and anthers, whereas lower activity was detected in the mature leaf, lemmas, and paleae (Figure 1B).

PEG4000 (simulated drought stress) and salt stresses upregulated the mRNA level of *OsTZF7*, and it reached the maximum level at 12 h in shoot and 3 h in root, respectively (Figure 1C). *OsTZF7* mRNA was also upregulated by ABA treatment but downregulated by cold stress.

### The Overexpression of *OsTZF7* Enhances Drought Tolerance in Rice

To identify whether *OsTZF7* contributes to drought tolerance in rice, transgenic rice plants with *OsTZF7* overexpression or knockdown (RNAi) were generated. Two overexpression lines (OE1 and OE4, > 50-fold) with an elevated expression and three RNAi lines (Ri5, Ri8, and Ri21, < 0.5-fold) with a decreased expression were used for further study.

**FIGURE 2:**
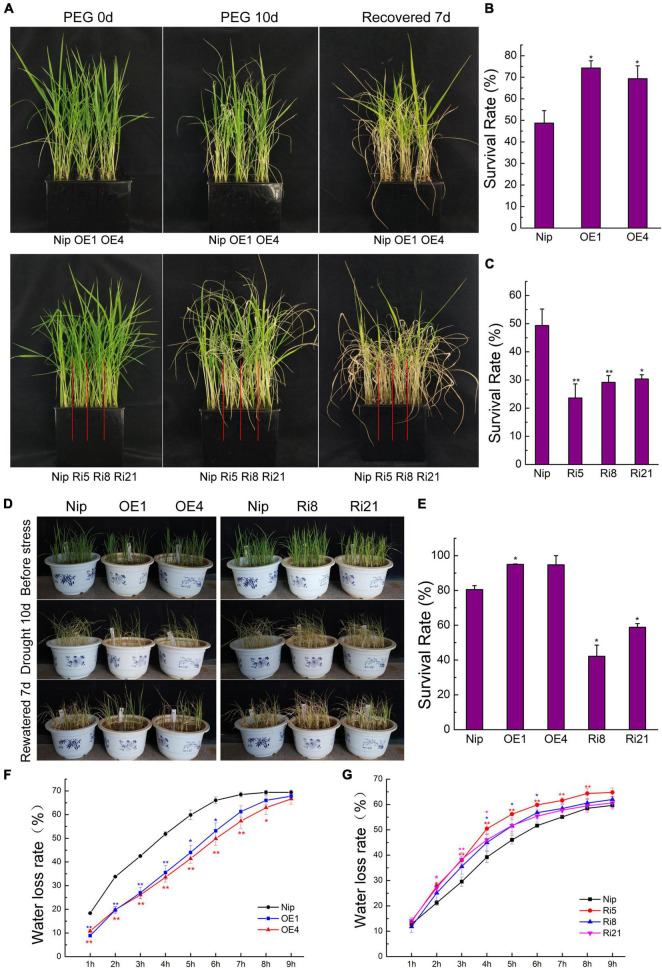
Phenotype of *OsTZF7* overexpressing and knockdown transgenic rice. **(A)** Performance of wild-type Nip, *OsTZF7*-OE, and RNAi plants before and after PEG treatment at the seedling stage. **(B,C)** The survival rates of Nip and *OsTZF7*-OE/RNAi seedlings after polyethylene glycol (PEG) treatment. **(D)** Performance of Nip, *OsTZF7*-OE, and RNAi seedlings before and after drought stress. **(E)** The survival rates of Nip, *OsTZF7*-OE, and RNAi seedlings after drought stress. **(F)** The water loss rate of detached leaves of Nip and *OsTZF7*-OE lines. **(G)** The water loss rate of detached leaves of Nip and *OsTZF7*-RNAi lines. Error bars indicate SE. Statistical significance is indicated by *P < 0.05; **P < 0.01, *t*-test.

Since *OsTZF7* mRNA was upregulated by PEG treatment, transgenic and wild-type plants were evaluated for drought tolerance. Rice seedlings at the four-leaf stage were grown in 20% PEG4000 (simulating drought stress) for 10 days and then were grown in Yoshida solution for 7 days to recover. After recovery, the survival rates of the OE lines OE1 and OE4 were 74.3 and 69.3%, respectively, whereas the survival rate of wild-type Nip plants was 48.7% (Figures 2A,B). In contrast, only 23–30% of RNAi plants recovered, which was significantly lower than wild-type Nip (49.3%) (Figures 2A,C). This result suggests that *OsTZF7* may play a positive role in drought resistance.

To further confirm the positive role of *OsTZF7* in drought resistance, we evaluated the drought tolerance of transgenic and wild-type plants at the vegetative stage. The vegetative-stage seedlings in barrels were subjected to drought stress by withdrawing water supply for 10 days, followed by rewatering for 7 days. As shown in Figures 2D,E, more than 94% of overexpression plants and 80.5% of Nip plants survived, while the survival rates of RNAi lines were less than 60%. In addition, we compared the water loss rate, which correlates with drought resistance, of detached leaves of transgenic and wild-type plants. Consistent with the analysis of drought tolerance, the OE lines lost water slower than the wild type did, whereas the water loss rates of RNAi lines were higher than that of wild type (Figures 2F,G). These results further supported that *OsTZF7* acts as a positive regulator in drought resistance.

### The Overexpression of *OsTZF7* Increases Abscisic Acid Sensitivity

**FIGURE 3:**
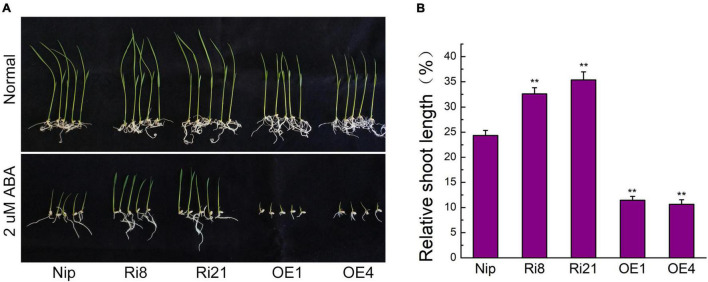
Effect of ABA treatment on seedling growth of Nip, *OsTZF7*-OE, and RNAi plants. **(A)** Growth performance of Nip, *OsTZF7*-OE, and RNAi plants subjected to 2 μM ABA on half-strength Murashige and Skoog (1/2 MS) medium. **(B)** Relative shoot length of Nip, *OsTZF7*-OE, and RNAi plants subjected to 2 μM ABA treatment. Error bars indicate SE. Statistical significance is indicated by **P < 0.01, *t*-test.

Since the expression of *OsTZF7* was upregulated by ABA treatment, we sought to determine the relationship between *OsTZF7* and ABA signaling. The OE lines showed a repressed growth (Figure 3A), while the RNAi lines showed improved growth under normal culture conditions at the seedling stage, which was similar to *OsTZF1* (Jan et al., 2013). Therefore, we used the relative shoot length of seedlings to evaluate the ABA sensitivity of transgenic plants. When grown on 1/2 MS supplemented with 2 μM ABA, the growth of *OsTZF7*-OE seedlings was severely suppressed (Figure 3A). The relative shoot length of *OsTZF7*-OE seedlings was significantly shorter than that of Nip plants (Figure 3B). In contrast, RNAi seedlings showed less sensitivity to ABA treatment (Figure 3A), and the relative shoot length of RNAi plants was longer than that of control (Figure 3B). These results indicated that *OsTZF7* may positively regulate ABA signaling.

### Transcriptome Profiling of *OsTZF7*-OE and RNAi Plants

**FIGURE 4:**
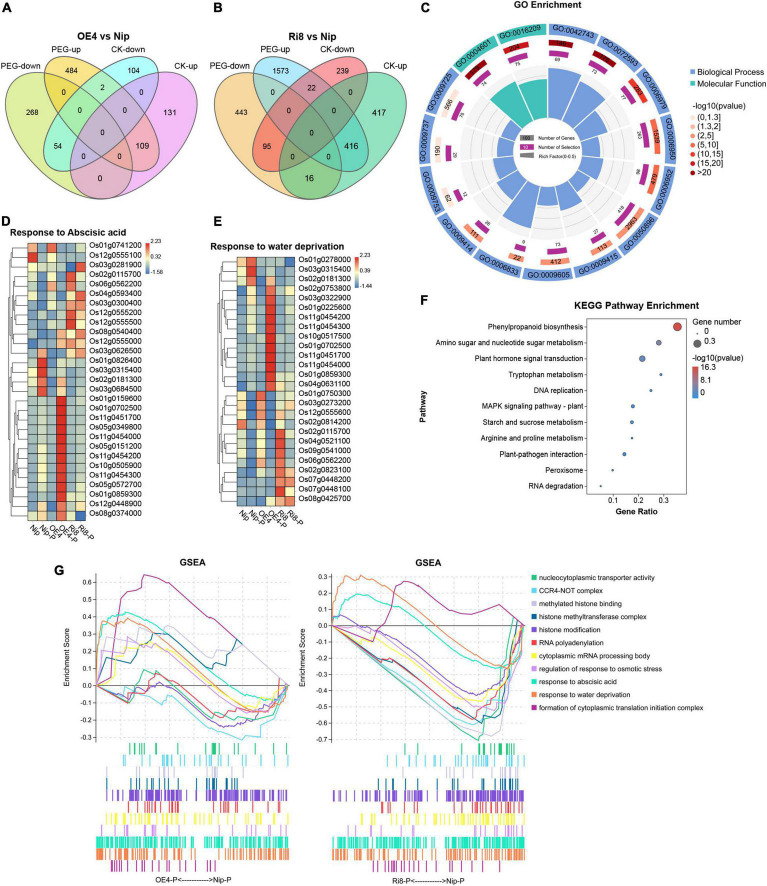
Transcriptome profiling of *OsTZF7* transgenic plants. **(A,B)** Venn diagram showing the number of genes regulated by *OsTZF7*-OE4 and Ri8 plants under normal or PEG conditions based on the RNA-seq analysis. **(C)** Significantly enriched Gene Ontology (GO) of all of the differentially expressed genes (DEGs). **(D,E)** Heatmaps showing the expression pattern of the genes related to “response to abscisic acid” **(D)**, “response to water deprivation” **(E)** in the Nip, *OsTZF7*-OE4, and Ri8 plants in response to PEG. **(F)** The Kyoto Encyclopedia of Genes and Genomes (KEGG) pathway enrichment analysis of all of the DEGs. **(G)** Dynamic GSEA analysis of Nip, *OsTZF7*-OE4, and Ri8 plants in response to PEG. All DEGs were determined by the threshold of log2(foldchange) ≥ 1 and false discovery rate (FDR) < 0.05 with three independent replicates.

To better understand the mechanism of *OsTZF7*-mediated drought tolerance, the transcriptomes of *OsTZF7*-OE4, *OsTZF7*-Ri8, and wild-type Nip plants under normal and PEG-simulated drought stress conditions were analyzed using RNA-seq. Compared to wild-type Nip, 160 and 849 genes were upregulated, and 240 and 356 genes were downregulated in OE4 and Ri8 lines under normal conditions, respectively, with a threshold of log2(foldchange) ≥ 1 (FDR < 0.05). When treated with PEG, 595 and 2011 genes were upregulated, and 322 and 554 genes were downregulated in OE4 and Ri8 lines, respectively (Figures 4A,B and Supplementary Table 2).

We selected 9 genes that were significantly changed in OE4 from the differentially expressed genes (DEGs) and performed qRT-PCR for verification. As shown in Supplementary Figure 1, when subjected to PEG treatment, the expression of stress-related genes Os01g0868000 (*OsEREBP2*), Os03g0230300 (*OsSRO1c*), Os10g0492600 (*OsTIP3*), Os03g0820300 (*ZFP182*), Os05g0542500 (*OsLEA3*), Os01g0702500 (dehydrin), and Os02g0513100 (*OsSWEET15*), MYB transcription factor Os02g0685200 were upregulated in OE lines, whereas no significant difference was observed between Nip and RNAi lines. A calmodulin gene, Os01g0949500 (*OsCML10*), was downregulated in OE lines under normal culture conditions, while no significant difference was observed between the transgenic and Nip plants under PEG stress conditions. Thus, OsTZF7 regulated stress-responsive genes during drought response in rice.

Then, we performed the GO enrichment analysis on all these DEGs. As shown in Figure 4C, several biological processes such as “response to abscisic acid (GO: 0009737),” “response to stress (GO: 0006950),” “response to water deprivation (GO: 0006833),” and “response to oxidative stress (GO: 0006979)” and molecular function such as “peroxidase activity (GO: 0004601)” were enriched. As shown in Figures 4D,E, the transcription level of ABA signaling-related bZIP transcription factor Os01g0859300 (*OsABI5*), dehydrin genes Os01g0702500, Os11g0451700, and Os11g0454000, late embryogenesis abundant (LEA) genes Os01g0159600 (*OsLEA1a*), Os01g0225600 (*OsLEA3-2*), Os03g0322900 (*RAB21*), and Os11g0454300 in *OsTZF7*-OE4 line were higher than wild-type Nip when treated with PEG, while no significant difference was observed between Ri8 line and wild-type Nip. In contrast, Os02g0115700 (*OsCATA*), aquaporin genes Os02g0823100 (*OsPIP1;3*), Os09g0541000 (*OsPIP2;7*), Os04g0521100, Os07g0448100, and Os07g0448200 were downregulated in the Ri8 plant. Furthermore, the Kyoto Encyclopedia of Genes and Genomes (KEGG) pathway enrichment analysis revealed that the DEGs were enriched in “Plant hormone signal transduction,” “MAPK signaling,” and “phenylpropanoid biosynthesis” pathways that may contribute to drought tolerance (Figure 4F).

The dynamic GSEA analysis was also performed to identify gene sets that may be associated with the phenotypes of *OsTZF7* transgenic plants. As shown in Figure 4G, the GO categories including “response to abscisic acid,” “response to water deprivation,” and “formation of cytoplasmic translation initiation complex” were positively regulated in the OE4 plant under PEG treatment. In addition, gene sets involved in “CCR4-NOT complex,” “methylated histone binding,” “histone methyltransferase complex,” “RNA polyadenylation,” and “cytoplasmic mRNA processing body” were negatively regulated and enriched in Ri8 seedling after PEG stress, while these gene sets were positively regulated or not enriched in OE4 plant.

### OsTZF7 Localizes in Both the Nucleus and Processing Bodies/Stress Granules, and the Leucine-Rich Nuclear Export Signal Mediates Its Nuclear Export

In mammalian cells, TZF proteins can localize in both the cytoplasmic foci and the nucleus and can rely on CRM1 for their export from the nucleus (Phillips et al., 2002; Bloch et al., 2011). To explore the subcellular localization of OsTZF7, transgenic rice expressing *OsTZF7*-*GFP* fusion driven by ubiquitin promoter was generated. As shown in Supplementary Figure 2A, the green fluorescence was predominantly observed in cytoplasm and PB- and SG-like cytoplasmic foci (arrows) in root cells at the young seedling stage. However, in root cells of the mature plant, GFP was exclusively found in the nuclei (Supplementary Figure 2B, arrows) but not in the cytoplasm or cytoplasmic foci, indicating that OsTZF7 might play different roles at different development stages.

**FIGURE 5:**
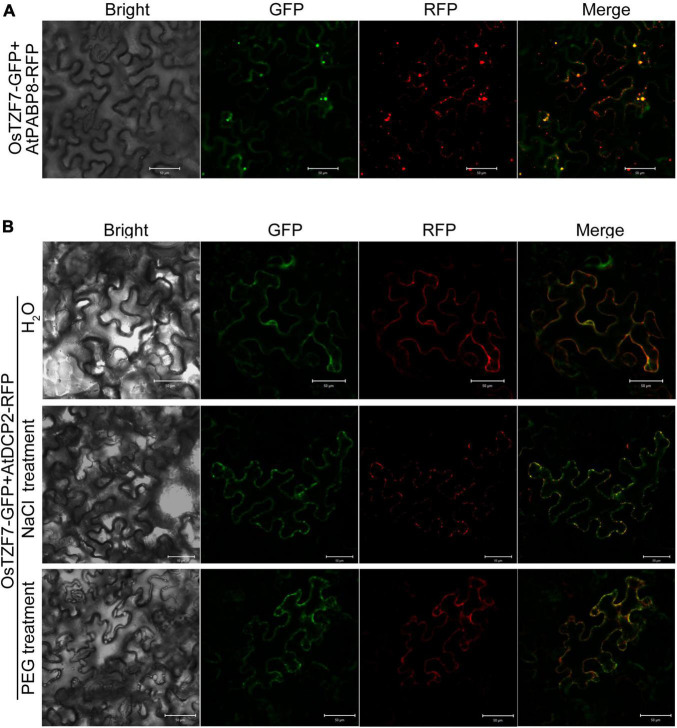
OsTZF7 colocalizes with processing bodies (PBs) and stress granules (SGs). **(A)** Colocalization of OsTZF7 with the SG marker AtPABP8. **(B)** Colocalization of OsTZF7 with the PB marker AtDCP2 under normal (top panel) or stress (middle and bottom panels) conditions. GFP, green fluorescent protein; RFP, red fluorescent protein. Scale bars = 50 μm.

To confirm the association of OsTZF7 with PBs and SGs, a colocalization analysis was performed by the co-expression of OsTZF7 with PB or SG makers from *Arabidopsis* or rice (Jan et al., 2013; Guo et al., 2016) in tobacco leaves. When co-expressed with *AtPABP8* or *OsPABP1*, *2*, *3*, which is the maker for SGs, OsTZF7 was found to colocalize with the PABPs in SG-like foci under normal conditions (Figure 5A and Supplementary Figure 3A). PB-like cytoplasmic foci were not obvious under normal conditions, and OsTZF7 and PB marker proteins AtDCP2 and OsDCP2 showed diffused expression pattern in the cytoplasm (Figure 5B, top panel. Supplementary Figure 3B, top panel). Given that PBs can be induced by stress, we cultured tobacco plants transfected with OsTZF7 and AtDCP2, or OsDCP2 with 150 mM NaCl (salt stress) or 20% PEG (stimulating drought stress) for 3 days. OsTZF7 colocalized with DCP2s in PB-like cytoplasmic foci in plants treated with NaCl or PEG (Figure 5B and Supplementary Figure 3B). Together, these results indicate that OsTZF7 can localize in both the nucleus and the cytoplasm, and the OsTZF7-associated cytoplasmic foci are induced by abiotic stress.

**FIGURE 6:**
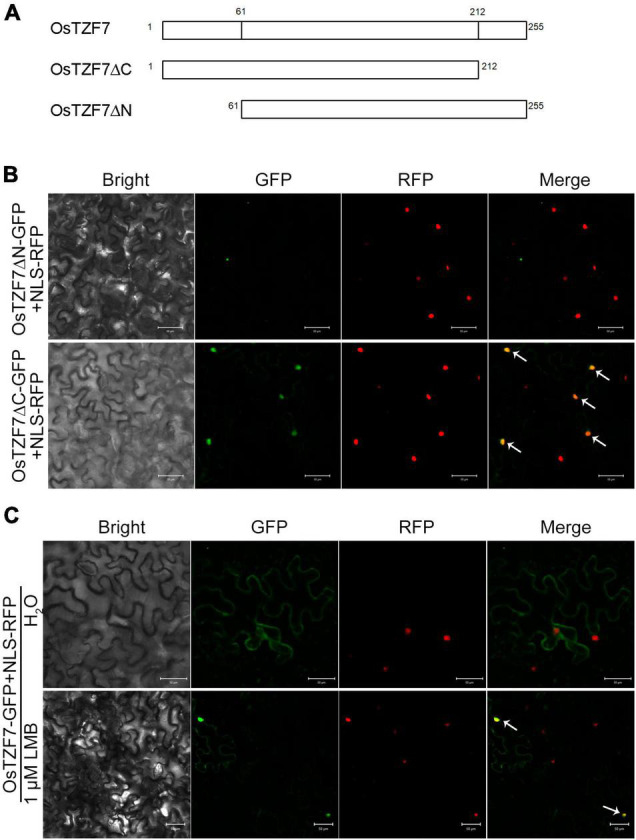
Leucine-rich nuclear export signal (NES) mediates the nuclear export of OsTZF7. **(A)** Schematic diagram of the OsTZF7 and the OsTZF7 mutant proteins (putative leucine-rich NES removed). **(B)** Tobacco leaves co-expressing OsTZF7-GFP mutants and NLS-RFP (OsTZF7ΔN, top panel; OsTZF7ΔC, bottom panel). **(C)** Tobacco leaves co-expressing OsTZF7-GFP and NLS-RFP treated with (bottom panel) or without (top panel) Leptomycin B (LMB). Arrows indicate nuclear OsTZF7. Scale bars = 50 μm.

To identify the nuclear export mechanism of OsTZF7, we analyzed the OsTZF7 protein sequence by NetNES prediction tool.^3^ The prediction result indicated that there were two putative leucine-rich NES in either N- or C-terminal region of OsTZF7. To determine which NES signal is responsible for the nuclear export of OsTZF7, we generated two OsTZF7-GFP mutants, OsTZF7ΔN-GFP (amino acids 1–60 removed) and OsTZF7ΔC-GFP (amino acids 213–255 removed), both driven by CaMV35s promoter (Figure 6A). An NLS-RFP construct was used to fluorescently label the nucleus. When wild-type OsTZF-GFP and NLS-RFP were co-expressed in tobacco leaves, GFP showed diffused expression pattern, and RFP was found exclusively in the nuclei (Figure 6C, top panel). In leaves transfected with OsTZF7ΔC-GFP and NLS-RFP, GFP and RFP were both found in the nuclei (Figure 6B, bottom panel), indicating that the C-terminal NES (amino acids 213–255) is necessary for the nuclear export of OsTZF7. In contrast, after removal of the N-terminal NES sequence, GFP were found in cytoplasmic foci (Figure 6B, top panel), indicating that N-terminal NES (amino acids 1–60) is not involved in nuclear export. In mammals, leucine-rich NES mediates the nuclear export of TTP or TIS11 by interacting with the nuclear export receptor CRM1 (Murata et al., 2002; Phillips et al., 2002). We next explored if CRM1 also mediates the nuclear export of OsTZF7. We treated tobacco leaves transfected with OsTZF7-GFP and NLS-RFP using Leptomycin B (LMB), a selective inhibitor of the CRM1 (Kudo et al., 1999), for 3–4 h. LMB treatment resulted in the nuclear accumulation of OsTZF7-GFP (Figure 6C, bottom panel). These results suggest that CRM1 might bind to the C-terminal NES and mediate the nuclear export of OsTZF7.

### OsTZF7 Binds AU-Rich and AU-Rich-Like Elements *in vitro*

**FIGURE 7:**
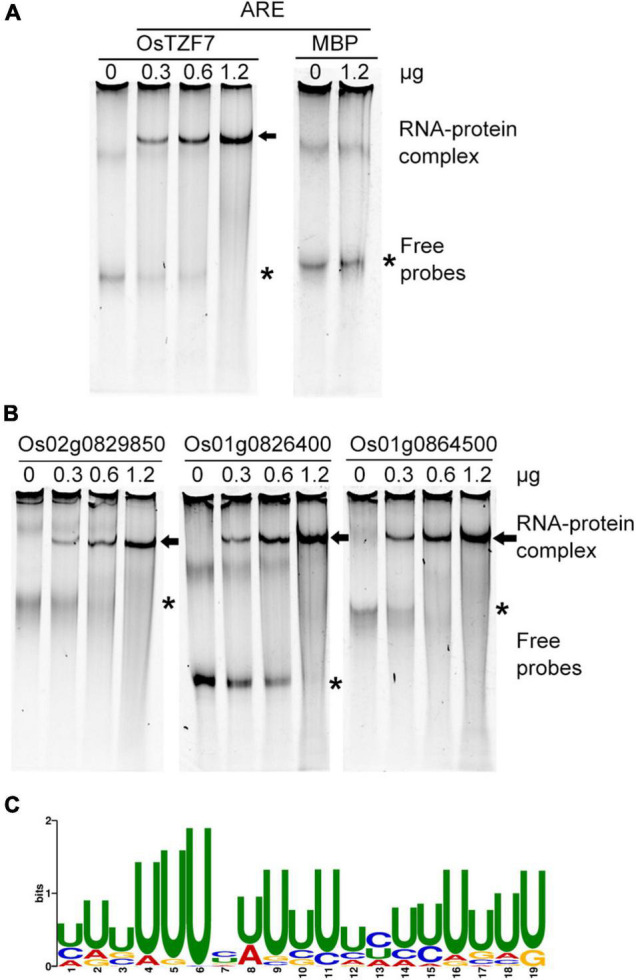
OsTZF7 binds to ARE and ARE-like motifs. **(A)** OsTZF7 bound to the ARE probe in the RNA electrophoretic mobility shift assay (REMSA). Maltose-binding protein (MBP) was used as a negative control. Arrow indicates shifted RNA-protein complex. Asterisk indicates free probes. **(B)** OsTZF7 bound to U-rich ARE-like sequences in the 3′UTR of Os02g0829850, Os01g0826400, and Os01g0864500 RNAs. **(C)** Discovered U-rich motif (*E*-value = 1.8e-025) within downregulated genes.

Since OsTZF7 was mainly localized in PBs and SGs (Figure 5 and Supplementary Figure 3), and most of the TZF proteins participate in posttranscriptional regulation by binding to AREs within the 3′UTR of target mRNAs in PBs and SGs (Otsuka et al., 2019), we hypothesized that OsTZF7 can also bind to AREs. To test this hypothesis, the REMSA was performed using recombinant OsTZF7 and *in vitro* transcribed ARE probe. We first purified 6 × His-SUMO-fused OsTZF7 using Ni-NTA Resin. Then, the on-column cleavage with Ulp1 was performed to remove the 6 × His-SUMO tag. The tag-free recombinant OsTZT7 was incubated with ARE probe at room temperature. As shown in Figure 7A, the recombinant OsTZT7 formed a complex with ARE probe and caused a shifted band on the gel. In contrast, no band shift was observed when ARE probe was incubated with maltose-binding protein (MBP) (Figure 7A).

Furthermore, we scanned all the downregulated genes in the *OsTZF7* overexpression plant for ARE motif (WWWWAUUUAUUUW) with the FIMO tool. Notably, 73 genes that contained ARE or ARE-like motifs within their 3′UTR were identified (Supplementary Table 3). The ARE-like motifs of three downregulated genes were selected and transcribed *in vitro*, and the REMSA was performed to determine the interaction between OsTZF7 and these ARE-like motifs. As shown in Figure 7B, band shifts were observed when the ARE-like motifs of Os02g0829850, Os01g0826400, and Os01g0864500 were incubated with recombinant OsTZF7. Moreover, the intensity of shifted probe increased with an increase in the amount of OsTZF7 used. We then used the MEME online tool to identify consensus motif within 3′UTR of all downregulated genes in the *OsTZF7* overexpression plant. A U-rich motif (*E*-value = 1.8e-025) was found to be enriched in these sequences (Figure 7C). Together, these results demonstrated that OsTZF7 can bind to ARE and ARE-like motifs *in vitro*.

### OsTZF7 Interacts With PWWP Proteins

**FIGURE 8:**
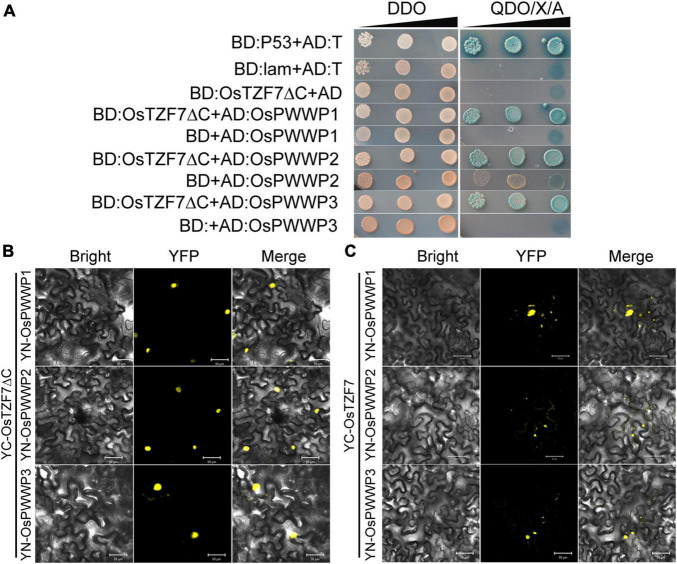
OsTZF7 physically interacts with OsPWWP1, 2, 3. **(A)** Yeast two-hybrid assays of OsTZF7 and OsPWWPs. SD, synthetic dropout medium. DDO, SD/-Leu-Trp. QDO/X/A, SD/-Ade-His-Leu-Trp/ + X-α-Gal/ + Aureobasidin A. **(B)** The bimolecular fluorescence complementation (BiFC) analysis indicated that YC-OsTZF7ΔC interacts with YN-OsPWWPs in tobacco leaves. **(C)** BiFC analysis indicated that YC-OsTZF7 interacts with YN-OsPWWPs in tobacco leaves. YFP, yellow fluorescent protein. Scale bars = 50 μm.

We used Y2H screening to identify the interacting proteins of OsTZF7, hoping to further elucidate the function of OsTZF7. We did not identify any interacting proteins using intact OsTZF7 as bait. We speculated that the NES signal at the C-terminal of OsTZF7 affects its nucleus localization. We then used OsTZF7ΔC (C-terminal NES removed) as bait for Y2H screening, and three putative interacting proteins (Os02g0700000, Os04g0599100, and Os01g0558500) were identified (Figure 8A). Interestingly, all three proteins belonged to the PWWP (Pro-Trp-Trp-Pro) family, and we here named them OsPWWP1, 2, 3.

To confirm the interaction between OsTZF7 and three PWWP proteins *in vivo*, we performed the BiFC assay in transiently transformed tobacco epidermal cells. As shown in Figures 8B,C and Supplementary Figure 4, OsTZF7ΔC interacted with OsPWWP1, 2, 3 in the nucleus, whereas the interaction between OsTZF7 and PWWP proteins was likely in both the nucleus and the cytoplasmic foci.

## Discussion

### *OsTZF7* Positively Regulates Drought Stress in Rice

Plant TZF proteins are known to participate in plant growth, development, and stress responses. In *Arabidopsis*, most of the AtTZFs have been well functionally characterized. *AtTZF1* enhances plant stress tolerance in an ABA- and GA-dependent manner (Lin et al., 2011). The overexpression of *AtC3H49/AtTZF3* or *AtC3H20/AtTZF2* confers ABA hypersensitivity and enhances drought tolerance (Lee et al., 2012). *AtTZF10* (*AtSZF2/AtC3H29*) and *AtTZF11* (*AtSZF1/AtC3H47*) positively regulate salt stress response (Sun et al., 2007). In rice, *OsTZF1*, *5*, *8* are involved in drought tolerance (Jan et al., 2013; Selvaraj et al., 2020; Seong et al., 2020). In this study, we identified OsTZF7 as a drought-responsive TZF protein. *OsTZF7* expression was upregulated by drought, salt stresses, and ABA (Figure 1C), suggesting that it may participate in drought stress regulation. When subject to PEG-simulated drought or drought stresses, *OsTZF7*-OE plants showed enhanced stress tolerance, and their detached leaves lost water slower than wild-type plants (Figure 2). In contrast, RNAi plants were more sensitive to drought stress and showed faster water loss (Figure 2), indicating that *OsTZF7* plays a positive role in rice drought tolerance.

The ABA as a stress hormone regulates plant abiotic stress response and triggers stomatal closure to reduce water loss. *OsTZF7* expression was upregulated by exogenous ABA, and the *OsTZF7*-OE seedlings showed hypersensitivity to exogenous ABA, whereas RNAi plants were insensitive to ABA (Figure 3). These results suggest that *OsTZF7* may be involved in ABA signaling. Furthermore, the RNA-seq analysis revealed that many stress-related genes are differentially expressed between wild-type and transgenic plants. Many of them were enriched in several abiotic stress-related biological processes including “response to water deprivation,” “response to abscisic acid,” “response to stress,” and “response to oxidative stress,” which could contribute to the enhanced drought tolerance. For example, ABA signaling-related *OsABI5*, dehydrin, and LEA genes were upregulated in the OE4 plant during PEG stress (Figure 4D). The KEGG enrichment analysis also showed that DEGs are enriched in “Plant hormone signal transduction” pathways (Figure 4F). These results implied that *OsTZF7* may regulate stress-related genes and enhance drought tolerance through the ABA-dependent pathway.

### OsTZF7 Binds to AU-Rich-Like Elements and May Be Involved in mRNA Turnover

The TZF proteins are shown dynamic subcellular localization patterns. They are found in the nucleus or PBs/SGs formed mRNP complexes, implying that TZF proteins may play different roles in different organelles. The mammalian TTP participates in PB- and SG-mediated posttranscriptional regulation including mRNA degradation and translation repression (Lykke-Andersen and Wagner, 2005; Anderson and Kedersha, 2009; Qi et al., 2012). In plants, AtTZF1 localizes in PBs and SGs, triggering the decay of ARE-containing mRNAs *in vivo* (Pomeranz M.C. et al., 2010; Qu et al., 2014). Furthermore, *AtTZF1* as a direct target is repressed by pseudo-response regulators (PRRs) and negatively regulates target of rapamycin (TOR) signaling by directly binding to the 3′UTR and triggering TOR mRNA degradation (Li et al., 2019). AtTZF9 is phosphorylated by PAMP-responsive MPK3 and MPK6 and may sequester and inhibit the translation of target mRNAs (Maldonado-Bonilla et al., 2014; Tabassum et al., 2020). OsTZF1 binds to the RNA of some downregulated genes containing U-rich and ARE-like motifs within their 3′UTR implying that OsTZF1 is involved in the RNA turnover process (Jan et al., 2013). We identified OsTZF7 as an RNA binding protein, which is predominately localized in PBs and SGs. In REMSAs, recombinant tag-free OsTZF7 bound to ARE motif and caused a band shift *in vitro* (Figure 7A). To address whether OsTZF7 is involved in the mRNA turnover of target genes, we searched for ARE motif in the downregulated genes of *OsTZF7* overexpression plants by the FIMO tool. Notably, 73 genes were found containing ARE or ARE-like motifs in their 3′UTR. OsTZF7 also bound ARE-like motifs within the mRNA of three downregulated genes (Figure 7B). We used the MEME tool to search the 3′UTR of downregulated genes in the *OsTZF7* overexpression plant for enriched consensus motif and found that a “U-rich” motif was enriched (Figure 7C). Therefore, these results suggest that OsTZF7 similar to most plant TZFs can bind ARE and U-rich motifs and may be involved in RNA turnover. Additional experiments such as cross-linking immunoprecipitation (CLIP)-seq and degradome-seq to identify target mRNA of OsTZF7 will gain insights into the RNA regulation mechanism of OsTZF7.

In addition, many TZFs have been found to localize in the nucleus and function as transcriptional regulators. Nuclear TTP acts as a corepressor of steroid nuclear receptors (i.e., ERα, PR, GR, and AR) in breast cancer cells (Barrios-Garcia et al., 2014, 2016). AtTZF7 (OXS2) activates floral integrator genes by binding to the BOXS2 elements in the promoters of target genes (Blanvillain et al., 2011). OsTZF9 (OsGZF1) regulates the accumulation of glutelins *via* the transcriptional inhibition of *GluB-1* by binding to the *GluB-1* promoter during grain development (Chen et al., 2014). Although OsTZF7 was localized in the nucleus of the mature root cells, both OsTZF7 and OsTZF7ΔC did not show the transcriptional activation activity in the autoactivation test of Y2H (data not shown). Interestingly, three PWWP domain-containing proteins, namely, Os02g0700000, Os04g0599100, and Os01g0558500, that interact with OsTZF7ΔC were identified by Y2H screening and then confirmed by BiFC (Figure 8). PWWP domain belongs to the Tudor domain “Royal family,” which consists of Tudor, plant Agenet, Chromo, PWWP, and MBT domains (Maurer-Stroh et al., 2003). The PWWP domain mainly functions as a chromatin methylation reader by recognizing both DNA and histone-methylated lysines (Rona et al., 2016). The function of OsPWWP1, 2, 3 has not been reported. Three *Arabidopsis* homologs of OsPWWPs, PWWP1, 2, and 3 are part of PEAT complexes that mediate histone deacetylation and heterochromatin condensation to facilitate heterochromatin silencing (Tan et al., 2018). PWO1 (PWWP1) recruits Polycomb Repressive Complex 2 (PRC2) to the chromatin by interacting with H3 through its PWWP domain and regulates *Arabidopsis* flowering and development (Hohenstatt et al., 2018). Besides, dynamic GSEA analysis indicated that OsTZF7 regulates the gene sets such as “histone methyltransferase complex” and “histone modification” (Figure 4G). Together, OsTZF7 may be involved in histone modification. But the exact function of OsTZF7 with PWWPs is still to be investigated in the future.

### C-Terminal Nuclear Export Signal Mediates the OsTZF7 Export From the Nucleus

Diverse or conflict results for the subcellular localization of plant TZF proteins were reported. For instance, AtTZF1 is found in the nucleus when ectopically expressed in onion epidermal cells (Han et al., 2014) but traffics between the nucleus and the cytoplasmic foci in maize protoplasts (Pomeranz M.C. et al., 2010). AtTZF7 (OXS2) is detected in both the nucleus and the cytoplasm in *Arabidopsis* cells (Blanvillain et al., 2011), and it is also found in cytoplasmic foci in etiolated maize mesophyll protoplasts (Pomeranz M. et al., 2010). AtTZF9 is mainly localized in cytoplasmic foci in *Arabidopsis* protoplasts, and AtTZF9-MAPK complexes are found in both the cytosol and the nucleus (Maldonado-Bonilla et al., 2014). These variable cellular distributions suggest that the dynamic localization of plant TZF proteins may be dependent on the experimental condition. In our research, OsTZF7-GFP was predominately detected in the cytoplasm and cytoplasmic foci in young root cells and observed in the nucleus of mature root cells (Supplementary Figure 1). Moreover, the colocalization experiment confirmed that OsTZF7 was associated with PBs and SGs (Figure 5), but the nuclear localization of OsTZF7 was not observed in tobacco leaves. This might be due to the different experimental conditions. In addition, PB/SG assembly is triggered by specific growth conditions and particular signal transduction pathways (Anderson and Kedersha, 2009).

Many TZF proteins are localized in the nucleus or the cytoplasmic foci. TTP, AtTZF1, 9, and OsTZF1 traffic between the nucleus and PBs/SGs (Phillips et al., 2002; Pomeranz M.C. et al., 2010; Jan et al., 2013; Maldonado-Bonilla et al., 2014). It has been reported that the majority of plant TZFs including OsTZF7 contain both NES and NLS sequences (Bogamuwa and Jang, 2014), but the nucleocytoplasmic trafficking mechanism of plant TZFs is still unknown. In mammals, CRM1 interacts with NES of TZF proteins TTP and TIS11 and mediates their export from the nucleus. We reasoned that OsTZF7 may export from the nucleus by the same mechanism. To address this hypothesis, we used a deletion study to identify the NES of OsTZF7, and the result showed that the C-terminal leucine-rich region is the functional NES (Figure 6B). We further demonstrated that OsTZF7 could shuttle between the nucleus and the cytoplasm since the inhibition of nuclear export receptor CRM1 using LMB resulted in the nucleus accumulation of OsTZF7 (Figure 6C). These results indicate that OsTZF7 is a nucleocytoplasmic trafficking protein and relies on the C-terminal NES for its nuclear export.

### OsTZF7 Working Model in Processing Bodies/Stress Granules

**FIGURE 9:**
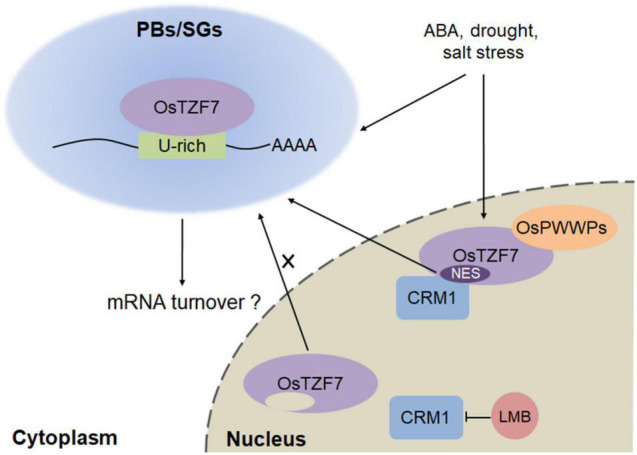
Working model of OsTZF7 in PBs/SGs during drought stress.

We revealed that OsTZF7 localizes in SG-like foci under normal culture conditions, while the salt and PEG treatment promote the accumulation of OsTZF7 in PB-like foci (Figure 5). These results are consistent with the previous reports that the formation of PBs is induced by stress (Motomura et al., 2015; Steffens et al., 2015). Therefore, our findings suggest that OsTZF7 may play important role in PBs/SGs, and we propose a simplified model for the roles of OsTZF7 in PBs/SGs during drought stress (Figure 9). In the nucleus, OsTZF7 interacts with OsPWWPs. CRM1 interacts with NES within the C-terminal of OsTZF7 and mediates the nuclear export of OsTZF7. The abrogation of CRM1 activity with LMB results in the nuclear accumulation of OsTZF7. Drought, salt stresses, and ABA upregulate the expression of *OsTZF7* and promote the aggregation of OsTZF7 in PBs/SGs. In PBs/SGs, OsTZF7 may regulate mRNA turnover by binding to the “U-rich” ARE-like motifs within the 3′UTR of target mRNAs and may enhance drought tolerance in rice.

## Data Availability Statement

The datasets presented in this study can be found in online repositories. The names of the repository/repositories and accession number can be found below: NCBI SRA, SRP342504.

## Author Contributions

CG and LC conceived the project, designed the experiments, and wrote the manuscript. CG and LLC conducted the main experiments. LL, YL, YY, and YG provided experimental assistance to CG and LLC. YC and MT helped with manuscript revision. All authors reviewed and approved the final manuscript.

## Conflict of Interest

The authors declare that the research was conducted in the absence of any commercial or financial relationships that could be construed as a potential conflict of interest.

## Publisher’s Note

All claims expressed in this article are solely those of the authors and do not necessarily represent those of their affiliated organizations, or those of the publisher, the editors and the reviewers. Any product that may be evaluated in this article, or claim that may be made by its manufacturer, is not guaranteed or endorsed by the publisher.

## Footnotes

1
http://wmd3.weigelworld.org/

2
www.omicshare.com/tools

3
https://services.healthtech.dtu.dk/service.php?NetNES-1.1

## References

[B1] Anderson P. Kedersha N. (2009). RNA granules: post-transcriptional and epigenetic modulators of gene expression. *Nat. Rev. Mol. Cell. Biol.* 10 430–436. 10.1038/nrm2694 19461665

[B2] Bailey T. L. Boden M. Buske F. A. Frith M. Grant C. E. Clementi L. (2009). MEME Suite: tools for motif discovery and searching. *Nucleic. Acids Res.* 37 W202–W208. 10.1093/nar/gkp335 19458158 PMC2703892

[B3] Balagopal V. Parker R. (2009). Polysomes, P bodies and stress granules: states and fates of eukaryotic mRNAs. *Curr. Opin. Cell Biol.* 21 403–408. 10.1016/j.ceb.2009.03.005 19394210 PMC2740377

[B4] Barrios-Garcia T. Gomez-Romero V. Tecalco-Cruz A. Valadez-Graham V. Leon-Del-Rio A. (2016). Nuclear tristetraprolin acts as a corepressor of multiple steroid nuclear receptors in breast cancer cells. *Mol. Genet. Metab. Rep.* 7 20–26. 10.1016/j.ymgmr.2016.02.004 27114912 PMC4832087

[B5] Barrios-Garcia T. Tecalco-Cruz A. Gomez-Romero V. Reyes-Carmona S. Meneses-Morales I. Leon-Del-Rio A. (2014). Tristetraprolin represses estrogen receptor alpha transactivation in breast cancer cells. *J. Biol. Chem.* 289 15554–15565. 10.1074/jbc.M114.548552 24737323 PMC4140911

[B6] Blanvillain R. Wei S. Wei P. Kim J. H. Ow D. W. (2011). Stress tolerance to stress escape in plants: role of the OXS2 zinc-finger transcription factor family. *EMBO J.* 30 3812–3822. 10.1038/emboj.2011.270 21829164 PMC3173794

[B7] Bloch D. B. Nobre R. A. Bernstein G. A. Yang W. H. (2011). Identification and characterization of protein interactions in the mammalian mRNA processing body using a novel two-hybrid assay. *Exp. Cell Res.* 317 2183–2199. 10.1016/j.yexcr.2011.05.027 21672539

[B8] Bogamuwa S. Jang J. C. (2013). The Arabidopsis tandem CCCH zinc finger proteins AtTZF4, 5 and 6 are involved in light-, abscisic acid- and gibberellic acid-mediated regulation of seed germination. *Plant Cell. Environ.* 36 1507–1519. 10.1111/pce.12084 23421766

[B9] Bogamuwa S. P. Jang J. C. (2014). Tandem CCCH zinc finger proteins in plant growth, development and stress response. *Plant. Cell Physiol.* 55 1367–1375. 10.1093/pcp/pcu074 24850834

[B10] Brooks S. A. Blackshear P. J. (2013). Tristetraprolin (TTP): interactions with mRNA and proteins, and current thoughts on mechanisms of action. *Biochim. Biophys. Acta* 1829 666–679. 10.1016/j.bbagrm.2013.02.003 23428348 PMC3752887

[B11] Carballo E. Lai W. S. Blackshear P. J. (1998). Feedback Inhibition of Macrophage Tumor Necrosis Factor-α Production by Tristetraprolin. *Science* 281 1001–1005. 10.1126/science.281.5379.1001 9703499

[B12] Chen Y. Sun A. Wang M. Zhu Z. Ouwerkerk P. B. (2014). Functions of the CCCH type zinc finger protein OsGZF1 in regulation of the seed storage protein GluB-1 from rice. *Plant Mol. Biol.* 84 621–634. 10.1007/s11103-013-0158-5 24282069

[B13] Fu M. Blackshear P. J. (2017). RNA-binding proteins in immune regulation: a focus on CCCH zinc finger proteins. *Nat. Rev. Immunol.* 17 130–143. 10.1038/nri.2016.129 27990022 PMC5556700

[B14] Guo C. Luo C. Guo L. Li M. Guo X. Zhang Y. (2016). OsSIDP366, a DUF1644 gene, positively regulates responses to drought and salt stresses in rice. *J. Integr. Plant Biol.* 58 492–502. 10.1111/jipb.12376 26172270

[B15] Han G. Wang M. Yuan F. Sui N. Song J. Wang B. (2014). The CCCH zinc finger protein gene AtZFP1 improves salt resistance in Arabidopsis thaliana. *Plant Mol. Biol.* 86 237–253. 10.1007/s11103-014-0226-5 25074582

[B16] Hohenstatt M. L. Mikulski P. Komarynets O. Klose C. Kycia I. Jeltsch A. (2018). PWWP-DOMAIN INTERACTOR OF POLYCOMBS1 Interacts with Polycomb-Group Proteins and Histones and Regulates Arabidopsis Flowering and Development. *Plant Cell* 30 117–133. 10.1105/tpc.17.00117 29330200 PMC5810566

[B17] Huang P. Chung M. S. Ju H. W. Na H. S. Lee D. J. Cheong H. S. (2011). Physiological characterization of the Arabidopsis thaliana oxidation-related zinc finger 1, a plasma membrane protein involved in oxidative stress. *J. Plant Res.* 124 699–705. 10.1007/s10265-010-0397-3 21188458

[B18] Huang P. Ju H. W. Min J. H. Zhang X. Chung J. S. Cheong H. S. (2012). Molecular and physiological characterization of the Arabidopsis thaliana Oxidation-related Zinc Finger 2, a plasma membrane protein involved in ABA and salt stress response through the ABI2-mediated signaling pathway. *Plant Cell Physiol.* 53 193–203. 10.1093/pcp/pcr162 22121246

[B19] Jan A. Maruyama K. Todaka D. Kidokoro S. Abo M. Yoshimura E. (2013). OsTZF1, a CCCH-tandem zinc finger protein, confers delayed senescence and stress tolerance in rice by regulating stress-related genes. *Plant Physiol.* 161 1202–1216. 10.1104/pp.112.205385 23296688 PMC3585590

[B20] Jefferson R. A. Kavanagh T. A. Bevan M. W. (1987). GUS fusions: beta-glucuronidase as a sensitive and versatile gene fusion marker in higher plants. *EMBO J.* 6 3901–3907. 3327686 10.1002/j.1460-2075.1987.tb02730.x PMC553867

[B21] Kim D. H. Yamaguchi S. Lim S. Oh E. Park J. Hanada A. (2008). SOMNUS, a CCCH-type zinc finger protein in Arabidopsis, negatively regulates light-dependent seed germination downstream of PIL5. *Plant Cell* 20 1260–1277. 10.1105/tpc.108.058859 18487351 PMC2438461

[B22] Kong Z. Li M. Yang W. Xu W. Xue Y. (2006). A novel nuclear-localized CCCH-type zinc finger protein, OsDOS, is involved in delaying leaf senescence in rice. *Plant Physiol.* 141 1376–1388. 10.1104/pp.106.082941 16778011 PMC1533915

[B23] Kudo N. Matsumori N. Taoka H. Fujiwara D. Schreiner E. P. Wolff B. (1999). Leptomycin B inactivates CRM1/exportin 1 by covalent modification at a cysteine residue in the central conserved region. *Proc. Nat. Acad. Sci. U.S.A.* 96 9112–9117. 10.1073/pnas.96.16.9112 10430904 PMC17741

[B24] Lai W. S. Blackshear P. J. (2001). Interactions of CCCH Zinc Finger Proteins with mRNA. *J. Biol. Chem.* 276 23144–23154. 10.1074/jbc.M100680200 11279239

[B25] Lee S. J. Jung H. J. Kang H. Kim S. Y. (2012). Arabidopsis zinc finger proteins AtC3H49/AtTZF3 and AtC3H20/AtTZF2 are involved in ABA and JA responses. *Plant Cell Physiol.* 53 673–686. 10.1093/pcp/pcs023 22383628

[B26] Li B. Wang Y. Zhang Y. Tian W. Chong K. Jang J. C. (2019). PRR5, 7 and 9 positively modulate TOR signaling-mediated root cell proliferation by repressing TANDEM ZINC FINGER 1 in Arabidopsis. *Nucleic. Acids Res.* 47 5001–5015. 10.1093/nar/gkz191 30892623 PMC6547441

[B27] Lin P. C. Pomeranz M. C. Jikumaru Y. Kang S. G. Hah C. Fujioka S. (2011). The Arabidopsis tandem zinc finger protein AtTZF1 affects ABA- and GA-mediated growth, stress and gene expression responses. *Plant J.* 65 253–268. 10.1111/j.1365-313X.2010.04419.x 21223390

[B28] Lin Y. J. Zhang Q. (2005). Optimising the tissue culture conditions for high efficiency transformation of indica rice. *Plant Cell Rep.* 23 540–547. 10.1007/s00299-004-0843-6 15309499

[B29] Livak K. J. Schmittgen T. D. (2001). Analysis of relative gene expression data using real-time quantitative PCR and the 2(-Delta Delta C(T)) Method. *Methods* 25 402–408. 10.1006/meth.2001.1262 11846609

[B30] Lykke-Andersen J. Wagner E. (2005). Recruitment and activation of mRNA decay enzymes by two ARE-mediated decay activation domains in the proteins TTP and BRF-1. *Genes Dev.* 19 351–361. 10.1101/gad.1282305 15687258 PMC546513

[B31] Maldonado-Bonilla L. D. (2014). Composition and function of P bodies in Arabidopsis thaliana. *Front. Plant Sci.* 5:201. 10.3389/fpls.2014.00201 24860588 PMC4030149

[B32] Maldonado-Bonilla L. D. Eschen-Lippold L. Gago-Zachert S. Tabassum N. Bauer N. Scheel D. (2014). The arabidopsis tandem zinc finger 9 protein binds RNA and mediates pathogen-associated molecular pattern-triggered immune responses. *Plant Cell Physiol.* 55 412–425. 10.1093/pcp/pct175 24285750

[B33] Mao X. Zhang H. Tian S. Chang X. Jing R. (2010). TaSnRK2.4, an SNF1-type serine/threonine protein kinase of wheat (Triticum aestivum L.), confers enhanced multistress tolerance in Arabidopsis. *J. Exp. Bot.* 61 683–696. 10.1093/jxb/erp331 20022921 PMC2814103

[B34] Maurer-Stroh S. Dickens N. J. Hughes-Davies L. Kouzarides T. Eisenhaber F. Ponting C. P. (2003). The Tudor domain ‘Royal Family’: Tudor, plant Agenet, Chromo, PWWP and MBT domains. *Trends Biochem. Sci.* 28 69–74. 10.1016/s0968-0004(03)00004-5 12575993

[B35] Motomura K. Le Q. T. Hamada T. Kutsuna N. Mano S. Nishimura M. (2015). Diffuse decapping enzyme DCP2 accumulates in DCP1 foci under heat stress in Arabidopsis thaliana. *Plant Cell Physiol.* 56 107–115. 10.1093/pcp/pcu151 25339350

[B36] Murata T. Yoshino Y. Morita N. Kaneda N. (2002). Identification of nuclear import and export signals within the structure of the zinc finger protein TIS11. *Biochem. Biophys. Res. Commun.* 293 1242–1247. 10.1016/s0006-291x(02)00363-7 12054509

[B37] Otsuka H. Fukao A. Funakami Y. Duncan K. E. Fujiwara T. (2019). Emerging Evidence of Translational Control by AU-Rich Element-Binding Proteins. *Front. Genet.* 10:332. 10.3389/fgene.2019.00332 31118942 PMC6507484

[B38] Phillips R. S. Ramos S. B. V. Blackshear P. J. (2002). Members of the Tristetraprolin Family of Tandem CCCH Zinc Finger Proteins Exhibit CRM1-dependent Nucleocytoplasmic Shuttling. *J. Biol. Chem.* 277 11606–11613. 10.1074/jbc.M111457200 11796723

[B39] Pomeranz M. Lin P.-C. Finer J. Jang J.-C. (2010). AtTZF gene family localizes to cytoplasmic foci. *Plant Signal. Behav.* 5 190–192. 10.4161/psb.5.2.10988 20173417 PMC2884132

[B40] Pomeranz M. C. Hah C. Lin P. C. Kang S. G. Finer J. J. Blackshear P. J. (2010). The Arabidopsis tandem zinc finger protein AtTZF1 traffics between the nucleus and cytoplasmic foci and binds both DNA and RNA. *Plant Physiol.* 152 151–165. 10.1104/pp.109.145656 19897605 PMC2799353

[B41] Qi M.-Y. Wang Z.-Z. Zhang Z. Shao Q. Zeng A. Li X.-Q. (2012). AU-Rich-Element-Dependent Translation Repression Requires the Cooperation of Tristetraprolin and RCK/P54. *Mol. Cell. Biol.* 32 913–928. 10.1128/MCB.05340-11 22203041 PMC3295194

[B42] Qu J. Kang S. G. Wang W. Musier-Forsyth K. Jang J. C. (2014). The Arabidopsis thaliana tandem zinc finger 1 (AtTZF1) protein in RNA binding and decay. *Plant J.* 78 452–467. 10.1111/tpj.12485 24635033 PMC4026020

[B43] Rona G. B. Eleutherio E. C. A. Pinheiro A. S. (2016). PWWP domains and their modes of sensing DNA and histone methylated lysines. *Biophys. Rev.* 8 63–74. 10.1007/s12551-015-0190-6 28510146 PMC5425739

[B44] Selvaraj M. G. Jan A. Ishizaki T. Valencia M. Dedicova B. Maruyama K. (2020). Expression of the CCCH-tandem zinc finger protein gene OsTZF5 under a stress-inducible promoter mitigates the effect of drought stress on rice grain yield under field conditions. *Plant Biotechnol. J.* 18 1711–1721. 10.1111/pbi.13334 31930666 PMC7336284

[B45] Seong S. Y. Shim J. S. Bang S. W. Kim J. K. (2020). Overexpression of OsC3H10, a CCCH-Zinc Finger, Improves Drought Tolerance in Rice by Regulating Stress-Related Genes. *Plants* 9:1298. 10.3390/plants9101298 33019599 PMC7599559

[B46] Shen Q. Liu Z. Song F. Xie Q. Hanley-Bowdoin L. Zhou X. (2011). Tomato SlSnRK1 protein interacts with and phosphorylates betaC1, a pathogenesis protein encoded by a geminivirus beta-satellite. *Plant Physiol.* 157 1394–1406. 10.1104/pp.111.184648 21885668 PMC3252149

[B47] Steffens A. Brautigam A. Jakoby M. Hulskamp M. (2015). The BEACH Domain Protein SPIRRIG Is Essential for Arabidopsis Salt Stress Tolerance and Functions as a Regulator of Transcript Stabilization and Localization. *PLoS Biol.* 13:e1002188. 10.1371/journal.pbio.1002188 26133670 PMC4489804

[B48] Sun J. Jiang H. Xu Y. Li H. Wu X. Xie Q. (2007). The CCCH-type zinc finger proteins AtSZF1 and AtSZF2 regulate salt stress responses in Arabidopsis. *Plant Cell Physiol.* 48 1148–1158. 10.1093/pcp/pcm088 17609218

[B49] Tabassum N. Eschen-Lippold L. Athmer B. Baruah M. Brode M. Maldonado-Bonilla L. D. (2020). Phosphorylation-dependent control of an RNA granule-localized protein that fine-tunes defence gene expression at a post-transcriptional level. *Plant J.* 101 1023–1039. 10.1111/tpj.14573 31628867

[B50] Tan L. M. Zhang C. J. Hou X. M. Shao C. R. Lu Y. J. Zhou J. X. (2018). The PEAT protein complexes are required for histone deacetylation and heterochromatin silencing. *EMBO J.* 37:e98770. 10.15252/embj.201798770 30104406 PMC6166130

[B51] Walter M. Chaban C. Schutze K. Batistic O. Weckermann K. Nake C. (2004). Visualization of protein interactions in living plant cells using bimolecular fluorescence complementation. *Plant J.* 40 428–438. 10.1111/j.1365-313X.2004.02219.x 15469500

[B52] Wang D. Guo Y. Wu C. Yang G. Li Y. Zheng C. (2008). Genome-wide analysis of CCCH zinc finger family in Arabidopsis and rice. *BMC Genom.* 9:44. 10.1186/1471-2164-9-44 18221561 PMC2267713

[B53] Xiang Y. Tang N. Du H. Ye H. Xiong L. (2008). Characterization of OsbZIP23 as a key player of the basic leucine zipper transcription factor family for conferring abscisic acid sensitivity and salinity and drought tolerance in rice. *Plant Physiol.* 148 1938–1952. 10.1104/pp.108.128199 18931143 PMC2593664

[B54] Zhang C. Zhang F. Zhou J. Fan Z. Chen F. Ma H. (2012). Overexpression of a phytochrome-regulated tandem zinc finger protein gene, OsTZF1, confers hypersensitivity to ABA and hyposensitivity to red light and far-red light in rice seedlings. *Plant Cell Rep.* 31 1333–1343. 10.1007/s00299-012-1252-x 22572927

[B55] Zhuang Y. Wang C. Zhang Y. Chen S. Wang D. Liu Q. (2020). Overexpression of PdC3H17 Confers Tolerance to Drought Stress Depending on Its CCCH Domain in Populus. *Front. Plant Sci.* 10:1748. 10.3389/fpls.2019.01748 32063912 PMC6999075

