# Supplementary material for: RNA Binding Protein OsTZF7 Traffics Between the Nucleus and Processing Bodies/Stress Granules and Positively Regulates Drought Stress in Rice
Source: Front Plant Sci. 2022 Feb 21;13:802337. doi: 10.3389/fpls.2022.802337 (PMC8899535; doi:10.3389/fpls.2022.802337)
Supplement: Supplementary file 4 [file Data_Sheet_1.pdf]

Supplementary Figures

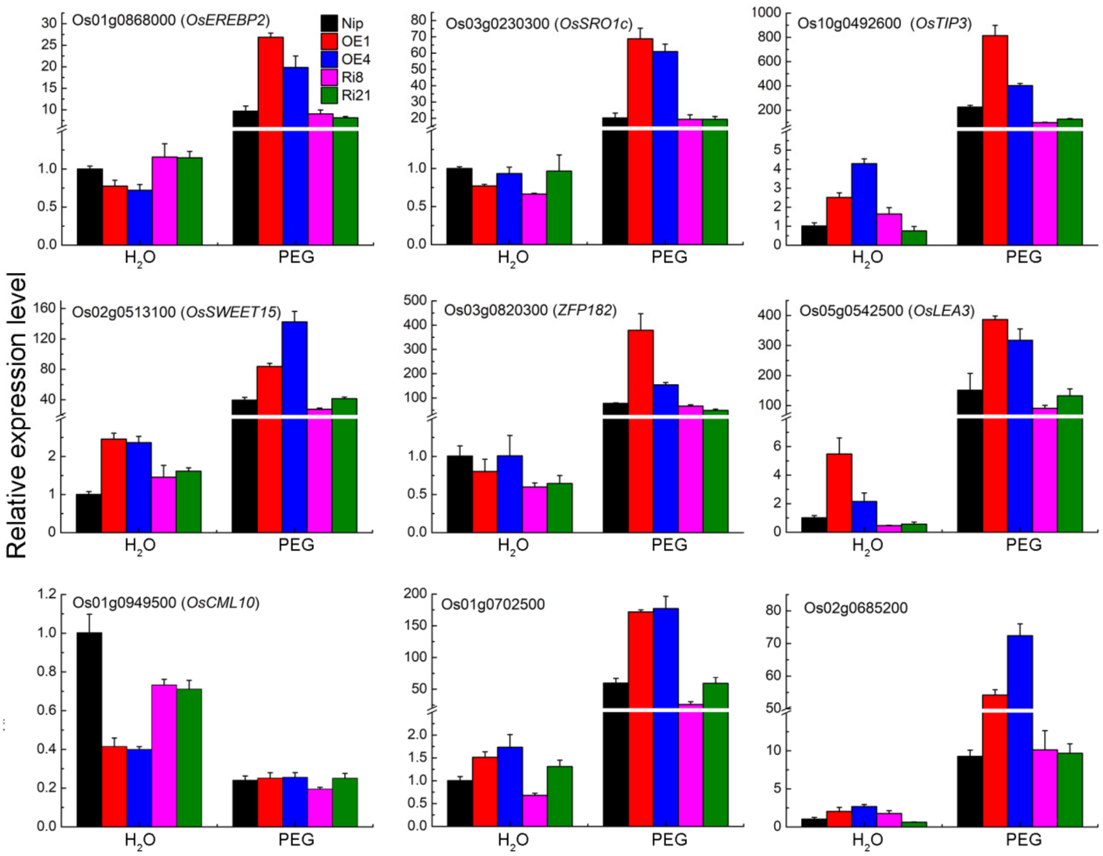

Supplementary Figure 1. Verification of the differentially expressed genes (DEGs) from RNA-seq analysis by qRT-PCR. Error bars indicate SD from three replicates.

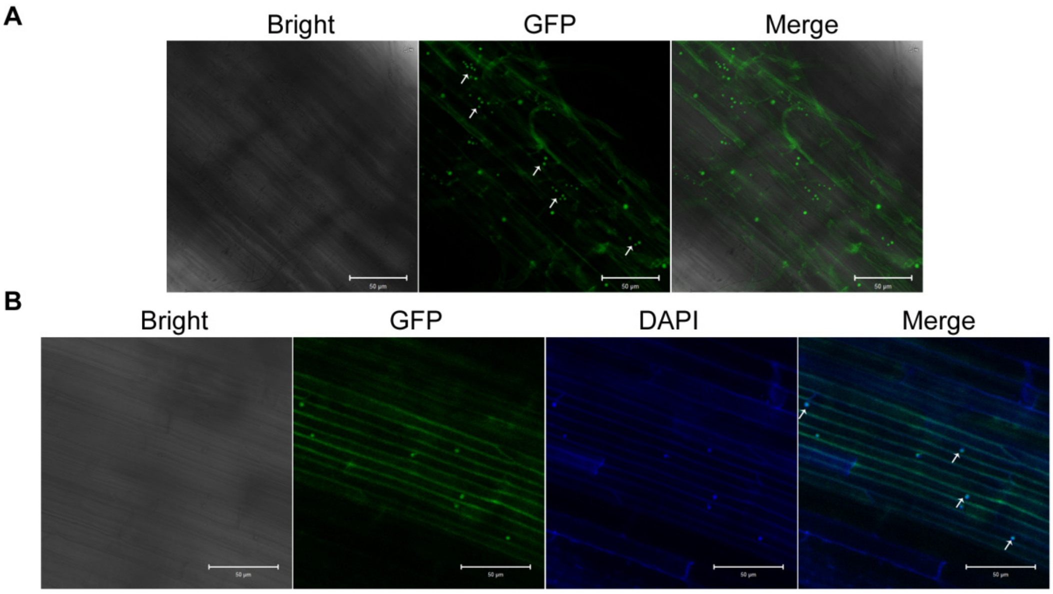

Supplementary Figure 2. Subcellular localization of OsTZF7. (A)#Subcellular localization of OsTZF7 in transgenic plants overexpressing the OsTZF7-GFP fusion protein, driven by the ubiquitin promoter. Arrow indicate cytoplasmic foci. (B) Nuclear localization of OsTZF7. GFP, green fluorescent protein. DAPI was used as a positive control for the nuclear localization. Arrow indicate the nucleus. Scale bars = 50 µm.

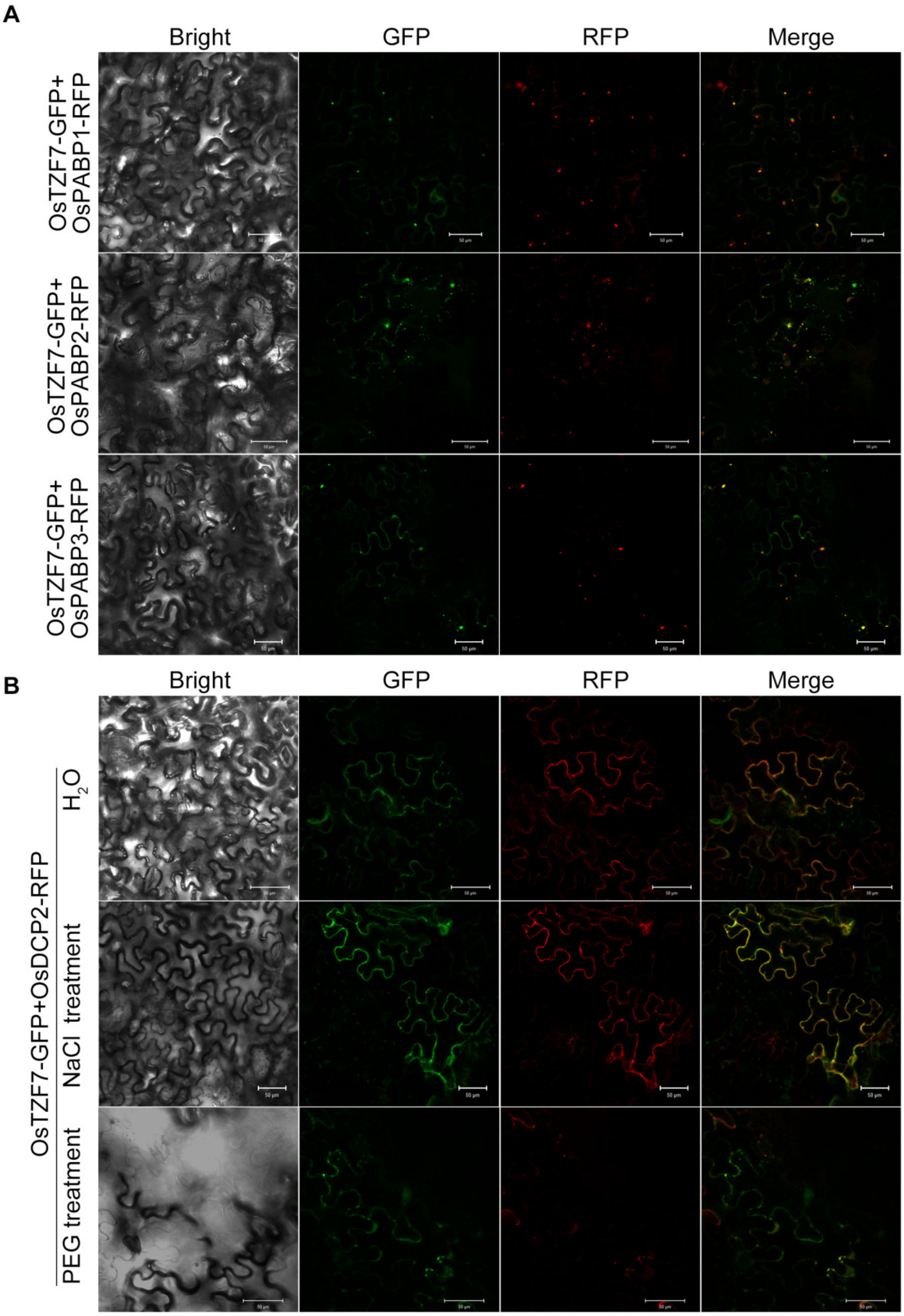

Supplementary Figure 3. OsTZF7 colocalizes with rice PBs and SGs marker.
(A) Colocalization of OsTZF7 with the rice SGs marker OsPABP1, 2, 3, respectively. (B) Colocalization of OsTZF7 with the rice PBs marker OsDCP2 under normal (top panel) and stress (middle and bottom panel) conditions. GFP, green fluorescent protein; RFP, red fluorescent protein. Scale bars = 50 μm.

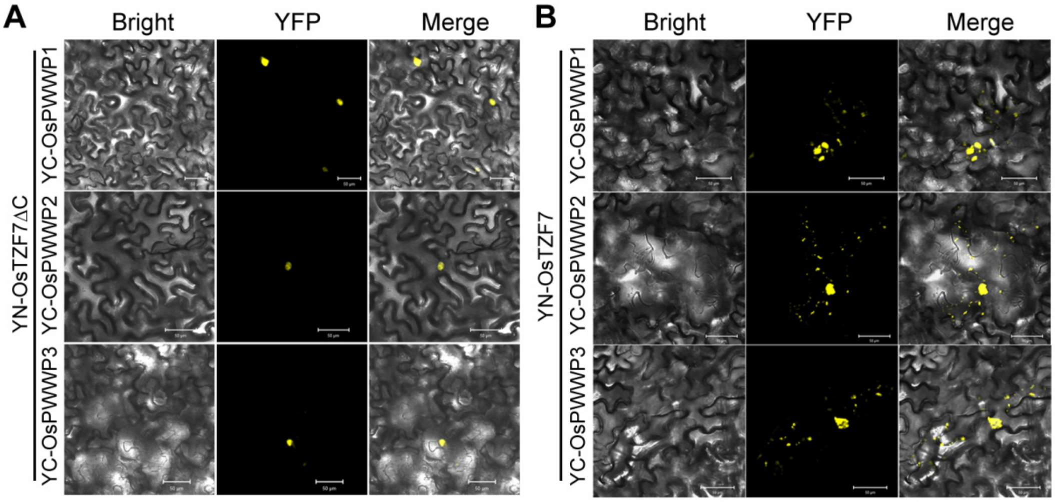

Supplementary Figure 4. BiFC analysis indicates that OsTZF7 or OsTZF7ΔC interacts with OsPWWPs in tobacco epidermal cells. (A) YN-OsTZF7ΔC+YC-OsPWWPs. (B) YN-OsTZF7+YC-OsPWWPs. YFP, yellow fluorescent protein. Scale bars = 50 μm.
